# Side Population in Human Non-Muscle Invasive Bladder Cancer Enriches for Cancer Stem Cells That Are Maintained by MAPK Signalling

**DOI:** 10.1371/journal.pone.0050690

**Published:** 2012-11-30

**Authors:** Anastasia C. Hepburn, Rajan Veeratterapillay, Stuart C. Williamson, Amira El-Sherif, Neha Sahay, Huw D. Thomas, Alejandra Mantilla, Robert S. Pickard, Craig N. Robson, Rakesh Heer

**Affiliations:** 1 Northern Institute for Cancer Research, Newcastle University, Framlington Place, Newcastle upon Tyne, United Kingdom; 2 Department of Urology, Freeman Hospital, The Newcastle upon Tyne Hospitals NHS Foundation Trust, Newcastle upon Tyne, United Kingdom; 3 Department of Pathology, Royal Victoria Infirmary, The Newcastle upon Tyne Hospitals NHS Foundation Trust, Newcastle upon Tyne, United Kingdom; 4 Institute of Cellular Medicine, Medical School, Newcastle University, Framlington Place, Newcastle upon Tyne, United Kingdom; The University of Texas M.D Anderson Cancer Center, United States of America

## Abstract

Side population (SP) and ABC transporter expression enrich for stem cells in numerous tissues. We explored if this phenotype characterised human bladder cancer stem cells (CSCs) and attempted to identify regulatory mechanisms. Focusing on non-muscle invasive bladder cancer (NMIBC), multiple human cell lines were used to characterise SP and ABC transporter expression. *In vitro* and *in vivo* phenotypic and functional assessments of CSC behaviour were undertaken. Expression of putative CSC marker ABCG2 was assessed in clinical NMIBC samples (n = 148), and a role for MAPK signalling, a central mechanism of bladder tumourigenesis, was investigated. Results showed that the ABCG2 transporter was predominantly expressed and was up-regulated in the SP fraction by 3-fold (ABCG2^hi^) relative to the non-SP (NSP) fraction (ABCG2^low^). ABCG2^hi^ SP cells displayed enrichment of stem cell markers (Nanog, Notch1 and SOX2) and a three-fold increase in colony forming efficiency (CFE) in comparison to ABCG2^low^ NSP cells. *In vivo*, ABCG2^hi^ SP cells enriched for tumour growth compared with ABCG2^low^ NSP cells, consistent with CSCs. pERK was constitutively active in ABCG2^hi^ SP cells and MEK inhibition also inhibited the ABCG2^hi^ SP phenotype and significantly suppressed CFE. Furthermore, on examining clinical NMIBC samples, ABCG2 expression correlated with increased recurrence and decreased progression free survival. Additionally, pERK expression also correlated with decreased progression free survival, whilst a positive correlation was further demonstrated between ABCG2 and pERK expression. In conclusion, we confirm ABCG2^hi^ SP enriches for CSCs in human NMIBC and MAPK/ERK pathway is a suitable therapeutic target.

## Introduction

Transitional cell carcinoma (TCC) of the urinary bladder has a worldwide annual incidence of over 350,000 new cases and 145,000 deaths [1]. In the US, it is the fifth most common malignancy accounting for over 70,000 new cases a year and an estimated annual spend of $3.5 billion on cancer treatment [2], [3]. Although 85% of patients present with less aggressive non-muscle invasive bladder cancer (NMIBC), they have a high risk of recurrence and those with unfavourable characteristics such as multifocal disease, high grade tumours, with or without concomitant carcinoma in situ (CIS), are at particular risk of disease progression. Intravesical Bacille Calmette-Guérin (BCG) can reduce the risk of progression by 27% in patients with high risk NMIBC [4] but those that fail to respond typically undergo morbid surgery to remove the bladder. For patients that present with, or progress to, muscle invasive bladder cancer (MIBC), the chance of surviving more than 5 years following treatment is approximately 50–60% [5], [6]. This background indicates that novel therapeutic approaches against NMIBC are urgently needed to eradicate disease before an invasive phenotype develops [7].

Recent advances in cancer biology have included the characterisation of cancer stem cells (CSCs), a small subpopulation of tumour cells that are tumour initiating and drive the production of the rest of the cancer. CSCs have been described in an increasing number of tumour sites including haematopoetic, breast, colon, melanoma and prostate [8]–[12]. It is becoming clear that it is essential to target these cells as they determine response to treatment [13]. The categorisation and selection of a side population (SP) phenotype by flow cytometry [14] enriches for CSCs in numerous tumours [15]–[18]. Recent demonstration of SP in bladder cancer necessitates further investigation of its potential role in characterising CSCs [19].

The pathogenesis of bladder cancer appears closely linked to distinct molecular pathways [20], [21]. Over 70% of NMIBCs harbour activating mutations of fibroblast growth factor receptor 3 (FGFR3) leading to constitutive activation of MAPK signalling [22], [23]. Furthermore, up-regulation of EGFR/ErbB family receptors is associated with increasing grade and stages of bladder cancer [24], [25]. Of relevance, SP phenotype is mediated by the ATP-binding cassette (ABC) family of transporter proteins [14], and the breast cancer-resistant protein-1 (BCRP1)/ABCG2 transporter has been shown to be the main mediator of this phenotype [26]. In particular, ABCG2 is in turn regulated by MAPK [27] and a role for MAPK/ERK signalling pathway in the regulation of SP has been suggested (Tsichuda et al 2008).

As a first step in informing the design of novel therapies for NIMBC, we aimed to characterise CSCs in human bladder cancer using SP selection and explore regulation through the MAPK/ERK signalling axis.

**Figure 1:**
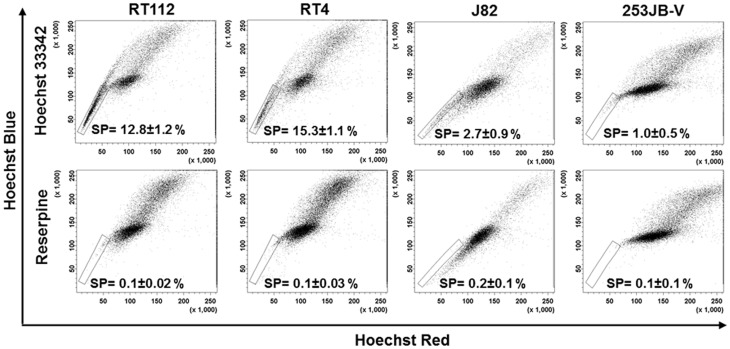
Identification of SP phenotype in bladder cancer cell lines. RT112, RT4, J82 and 253JB-V cells were stained with Hoechst 33342 dye alone or in the presence of reserpine and analysed by flow cytometry measuring Hoechst blue vs Hoechst red fluorescence. The SP was gated and represented as a percentage of the whole viable cell population following PI exclusion (mean ±SE).

**Figure 2:**
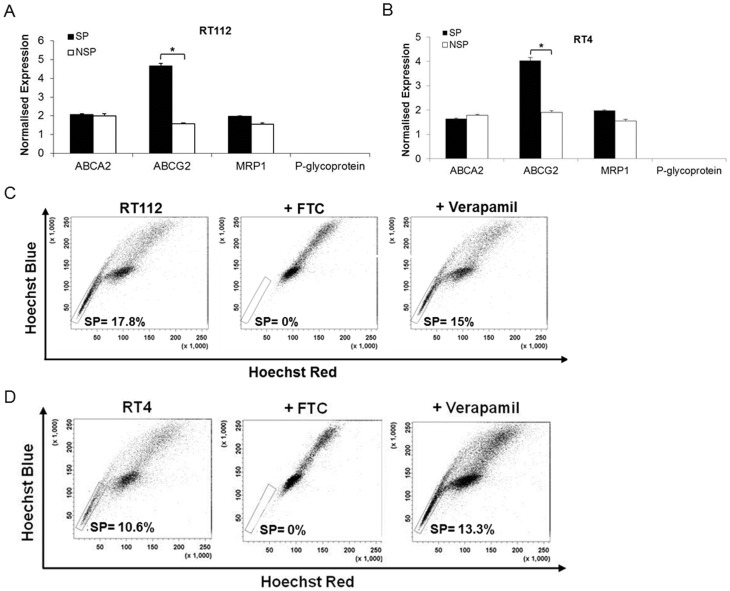
NMIBC SP cells show elevated ABCG2 transporter expression. Relative mRNA expression of ABCA2, ABCG2, MRP1 and P-glycoprotein was determined in SP and NSP fractions of RT112 (A) and RT4 (B) cells using real time PCR. Data shown are the mean of three independent experiments each performed in triplicate (mean ±SE). *P<0.05 (Student’s two-tailed *t*-test). Inhibition studies were performed on RT112 (C) and RT4 (D) cells using ABCG2-specific inhibitor fumitremorgin C (FTC) and P-glycoprotein inhibitor verapamil.

## Materials and Methods

### Reagents and Cell culture

Human bladder cancer cell RT4, RT112 and J82 were obtained from the American Type Culture Collection (ATCC) and cultured in RPMI 1640 (Sigma) supplemented with 10% fetal bovine serum (FBS) and 1% L-glutamine - referred to as full media (FM). The highly metastatic human TCC cell line 253JB-V was generously provided by Prof Colin Dinney [28] and cultured in MEMalpha media supplemented with 5% FBS. All cells were grown at 37°C in the presence of 5% CO_2_. MEK1/2 specific inhibitor, U0126 (Cell Signalling Technology), and rEGF (Sigma) were utilised in studies to modulate MAPK signalling. Control cells for comparison included cultures in basal media (BM) lacking FBS and FM.

### Hoechst Dye Efflux Assay

Cells were stained with Hoechst 33342 dye using modification of a previously described protocol [14]. Briefly, Hoechst 33342 dye (2.5 µg/ml) was added alone or in the presence of reserpine (50 µM), verapamil (50 µM) or fumitremorgin C (10 µM) and cells were incubated at 37°C for 90 min and shaken every 30 min. Cell surface marker analyses following Hoechst 33342 dye staining were performed with anti-ABCG2-FITC (clone 5D3, Millipore) co-labelling on ice for 20 min. Normal mouse IgG-FITC was used as isotype control (Upstate). Propidium iodide (PI) (2 µg/ml) gated viable cells. Analysis and sorting were carried out on a FACSDIVA flow cytometer (Becton Dickinson).

### RNA Extraction and Real-time PCR Analysis

Total RNA was extracted using the High Pure RNA kit (Roche) and reversed transcribed using random primers (Promega) and Superscript III reverse transcriptase enzyme (Invitrogen). Real time PCR was carried out with SYBR Green mastermix containing Rox™ and UDG (Invitrogen) using the ABI Prism 7900HT Sequence Detection System (Applied Biosystems). For primer sequences see Table S1. Levels of expression were normalised to GAPDH housekeeping gene.

**Figure 3:**
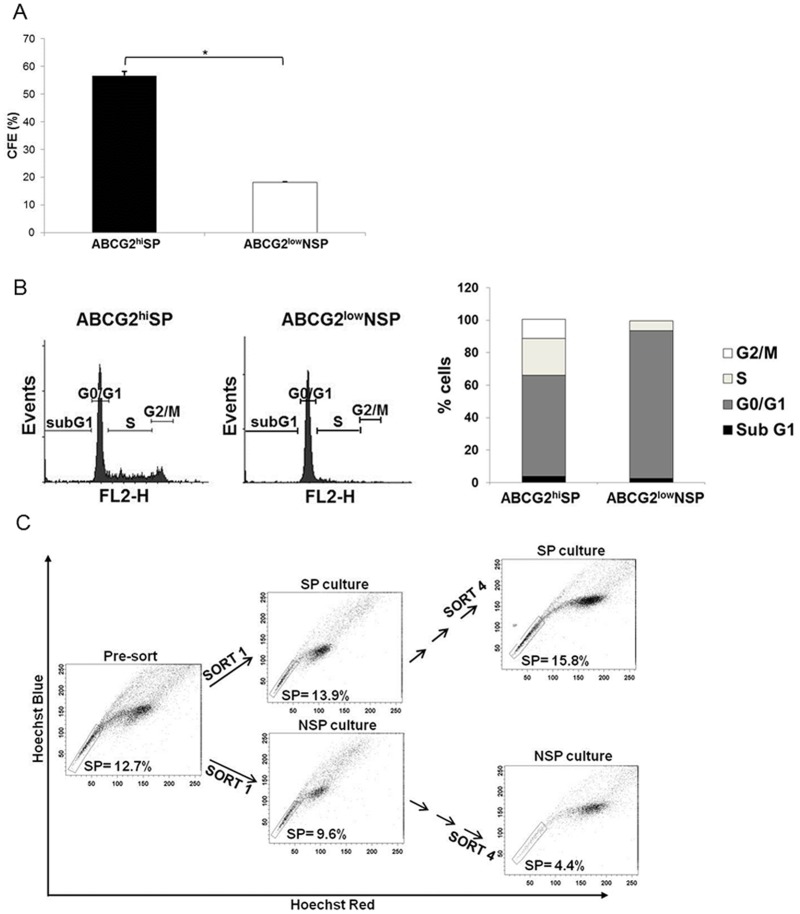
ABCG2^hi^ SP cells have a higher clonogenic ability in comparison to ABCG2^low^ NSP cells. (A) FACS sorted cells were seeded in 6-well plates at a density of 1×10^2^ cells/well and colonies were counted after 2 weeks. Colony forming efficiency (CFE) was calculated as described in Materials and Methods. Data shown are the mean (±SE) of three independent experiments each done in triplicate. *P<0.05 (Student’s two-tailed *t*-test). (B) Cell cycle profiles of RT112 ABCG2^hi^ SP and ABCG2^low^ NSP cells showing an increase in S-phase in the ABCG2^hi^ SP fraction. (C) Serial selection, sub-culture and cytometric fractioning between RT112 SP and NSP cells. SP and NSP cells sorted from RT112 were cultured for two weeks, restained with Hoechst 33342 dye and reanalysed by flow cytometry. This process was repeated 4 times.

**Figure 4:**
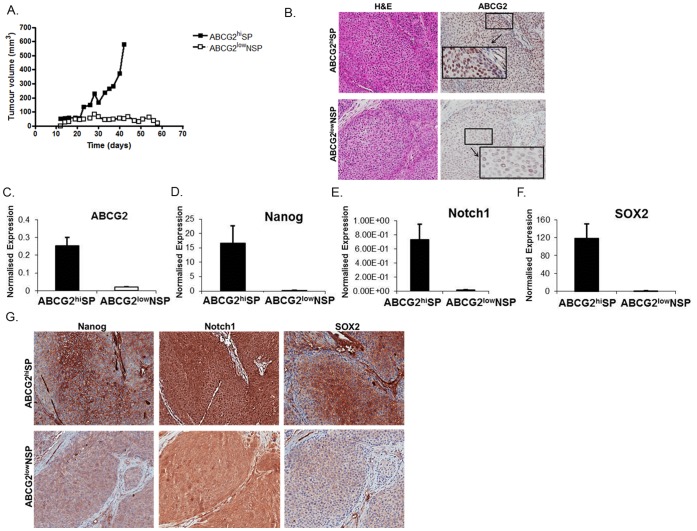
SP cells are more tumourigenic. (A) 1×10^3^ ABCG2^hi^ SP and ABCG2^low^ NSP sorted RT112 cells were subcutaneously injected into nude mice and monitored for tumour growth. (B) Immunohistochemical staining of serial sections, of tumours derived from mice injected with 1×10^3^ ABCG2^hi^ SP and residual cell pellets from mice injected with 1×10^3^ ABCG2^low^ NSP sorted RT112 cells, with H&E and ABCG2 and at higher magnification (20x). Markedly increased immunoreactivity for ABCG2 is seen in the ABCG2^hi^SP derived tumours. (C) Relative mRNA expression of ABCG2 in tumours formed following injection of 1×10^3^ ABCG2^hi^ SP and ABCG2^low^ NSP sorted RT112 cells was determined using real time PCR. (D–F) Relative mRNA expression of Nanog, Notch1 and SOX2 in tumours formed following injection of 1×10^3^ ABCG2^hi^ SP and ABCG2^low^ NSP sorted RT112 cells was determined using real time PCR. (G) Immunohistochemical staining of serial sections, of tumours formed following injection of 1×10^3^ ABCG2^hi^ SP and ABCG2^low^ NSP sorted RT112 cells, with Nanog, Notch1 and SOX2.

**Table 1:** Tumourigenesis of ABCG2^hi^SP and ABCG2^low^NSP cells in nude mice at 30 days.

Cell Number Injected	ABCG2^hi^SP	ABCG2^low^NSP
**100,000**	3/3	–
**10,000**	5/5	5/5
**1,000**	5/5	0/5

### Colony Forming Assay

FACS sorted cells were seeded into six-well plates at a density of 100 cells/well. After 14 days colonies were fixed with Carnoy’s fixative and stained with 0.4% crystal violet (w/v) for colony counting using ColCounter (Oxford Optronix), omitting <64 cell colonies as they represent early abortive colonies. Colony forming efficiency (CFE, %) was calculated as [(no. colonies counted/no. cells seeded) × 100].

### Cell Cycle

FACS sorted cells were stained with DAPI for cell cycle analysis using CyStain DNA 2step kit (Partec). Propidium iodide (2 µg/ml) gated viable cells. To discriminate against nuclear debris and doublets, cells were gated on FSC and SSC. Flow cytometry cell cycle software (CellQuest Pro, BD Biosciences) was used to calculate DNA content and calculate subG1, G1, S-phase and G2M phase statistics.

### *In vivo* Tumourigenicity

Animal experiments were performed and conducted in accordance to regulatory guidelines. Twenty three female athymic CD1 nude mice (Charles River, UK) were injected with RT112 SP and NSP sorted cells into the right flank subcutaneous tissue. Tumour growth was monitored by two dimensional measurement with electronic callipers with tumour volume calculated using the formula *a^2^*×*b/2*, where *a* is the smallest measurement and *b* the largest. The mice experiments were terminated when tumours grew to a maximum of 750 mm^3^. The tumours were removed and halved for immunohistochemical studies and real time PCR studies.

### Immunohistochemitry

Formalin fixed paraffin embedded archival material from patients undergoing endoscopic resection of bladder tumour or radical cystectomy was accessed for immunohistochemical studies with appropriate informed consent and ethical regulatory approval. In total, 148 NMIBC cases were stained with anti-ABCG2 (1∶250; clone BXP-21, Millipore) and anti-p-ERK (1∶100, E-4, Santa Cruz Biotechnology) before incubation with secondary biotinylated goat anti-mouse IgG antibody (DAKO). Immunoreactivity was visualised using Vectastain Avidin Biotin Complex Kit (Vector Laboratories) and 3′3′-diaminobenzidetetrahydrochloride. Scoring was initially assessed by percentage and stain intensity. However, the vast majority of cores expressed uniform epithelial staining with a single intensity, with heterogeneous staining present in only a few cases. In these cores, the intensity with >50% area of staining was taken as the score. The immunostaining was reviewed and scored independently by three assessors that were blinded to the clinical data to give average scores of staining intensity of absent (0), weak (1), moderate (2) or strong (3). Xenografts were also stained with anti-Nanog (1∶5000, Cell Signalling), anti-Notch 1 (1∶500, C-20, Santa Cruz Biotechnology) and anti-SOX2 (1∶500, Millipore).

### Western Blotting

Cells were lysed in SDS sample buffer (0.125 M Tris pH 6.8, 2% SDS, 10% glycerol, 10% β-mercaptoethanol and 0.01% bromophenol blue) and analysed by SDS-PAGE on 12% polyacrylamide gels, followed by transfer to nitrocellulose Hybond™ membrane (Fisher Scientific). Antibodies were used at the following dilutions: anti-ERK 1∶500 (K-23, Santa Cruz Biotechnology) and anti-phospho-ERK 1∶500 (E-4, Santa Cruz Biotechnology). Horseradish peroxidase-conjugated secondary antibodies (DAKO) were used at 1∶500 and detected using the enhanced chemiluminescence detection kit (Fisher Scientific).

### Statistical Analysis

For real time PCR studies, the two-tailed paired *t*-test was used to determine statistical significance at a level of P<0.05. For immunohistochemistry, differences in the frequency and expression of ABCG2 were examined using Mann-Whitney *U* test to assess correlations with pathological cancer grade and stage. Patient survival was analysed using the Kaplan-Meier method with log-rank testing, and multivariate analysis was performed using the Cox proportional hazards model. Pearson’s correlation was used to correlate ABCG2 and pERK expression. All tests were undertaken using SPSS version 11.0 computer software (SPSS, Inc.). All tests were two-sided and a *P* value of <0.05 was taken to indicate statistical significance.

**Figure 5:**
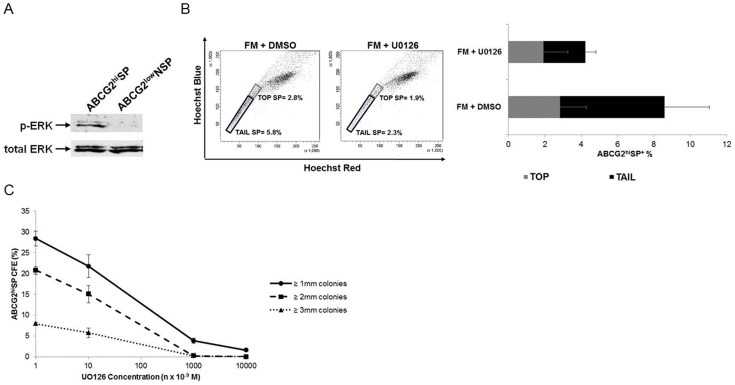
Inhibition of ERK signalling attenuates the fraction of ABCG2^hi^ SP RT112 cells. (A) Western blot analysis was performed with phospho-ERK1/2 and ERK1/2 antibodies. (B) RT112 cells were placed in FM alone or in the presence of U0126 (10 µM) for 30 min prior to staining with Hoechst 33342. The effect of U0126 on the ‘tail’ and ‘top’ end compartments of ABCG2^hi^SP^+^ was investigated. (C) CFE of ABCG2^hi^ SP cells with increasing concentrations of UO126 and different sized colonies (≥1 mm, ≥2 mm and ≥3 mm in diameter).

**Figure 6:**
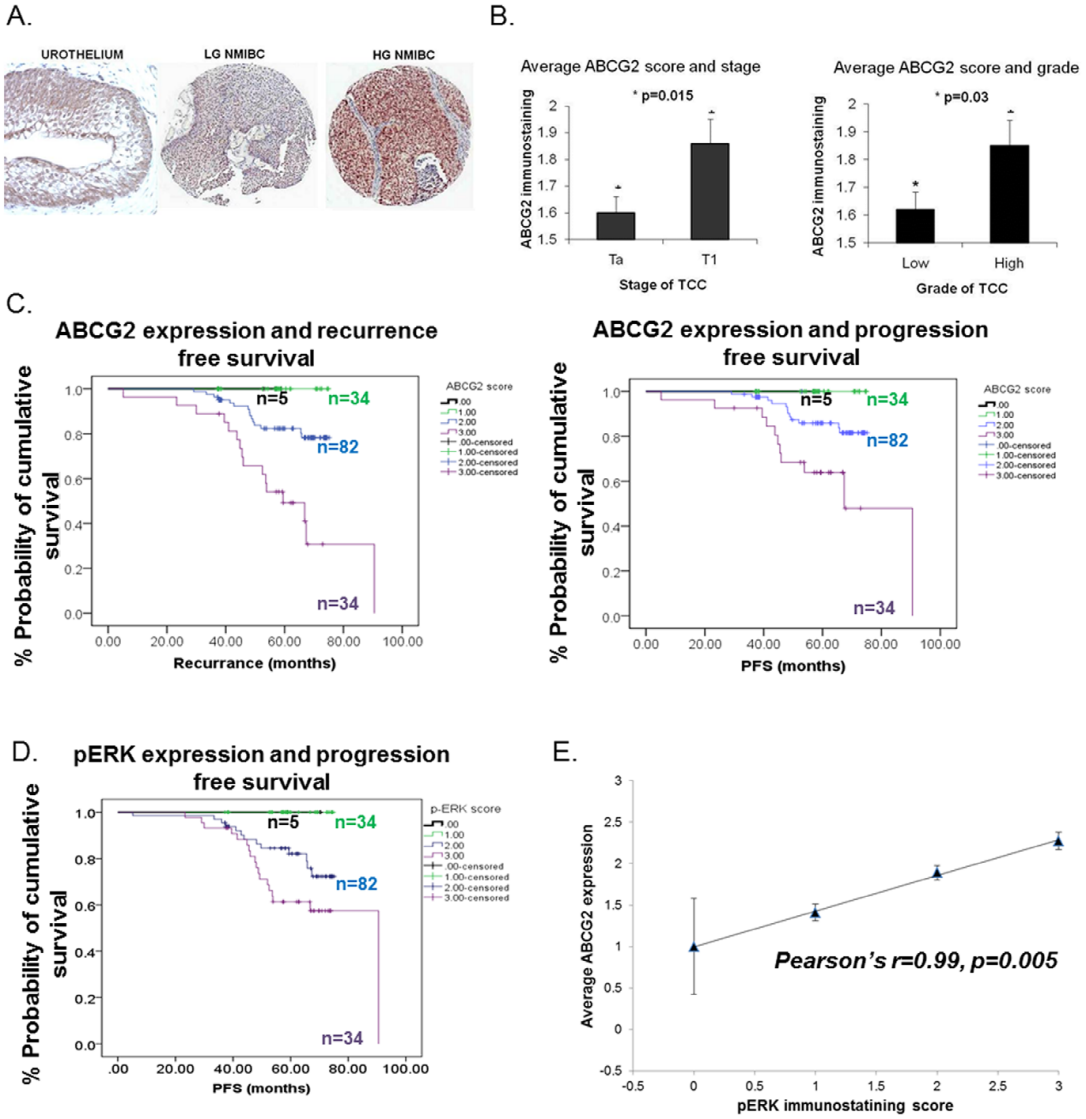
Immunohistochemical expression of ABCG2 in clinical bladder cancer. (A) ABCG2 expression in normal urothelium, low grade (LG) and high grade (HG) NMIBC is typically non-specific and can be seen nuclear and cytoplasmic. (B) Correlation of ABCG2 immunostaining intensity with grade and stage in NMIBC. (C) Kaplan-Meier curves illustrating outcomes of ABCG2 intensity (0, 1, 2 and 3) with recurrence and progression free survival (p<0.001, Log Rank analyses). (D) Kaplan-Meier curves illustrating outcomes of pERK intensity (0, 1, 2 and 3) with progression free survival (p<0.001, Log Rank analyses). (E) Correlation of ABCG2 and pERK expression (p = 0.005, Pearson’s r = 0.99).

## Results

### SP Cells and ABC Transporter Expression can be Identified in Human Bladder Cancer Cells

We investigated the presence of SP in two NMIBC cell lines (RT112 and RT4) and two MIBC cell lines (J82 and 253JB-V). Following staining with Hoechst 33342, a SP was identified in all four cell lines as a distinct tail extending from the main population with the characteristic low fluorescent profile in dual wavelength analysis (Figure 1). The gating strategy undertaken was followed as described by Golebiewska et. al. [29] (Figure S1). Appropriate discrimination of single and viable cells is crucial for adequate SP characterisation. The median (±SE) proportion of each total cell population accounted for by the SP was 12.8% (±1.2%) in RT112, 15.3% (±1.1%) in RT4, 2.7% (±0.9%) in J82, and 1.0% (±0.5%) in 253JB-V. The pan-ABC transporter inhibitor reserpine verified SP phenotype by blocking Hoechst efflux and inhibiting low red/blue staining phenotype that characterises SP cells.

We assessed ABC transporter expression patterns of bladder cancer cell lines using real time PCR. Heterogeneous expressions of multiple multidrug resistant genes were demonstrated in the MIBC cell lines J82 and 253JB-V. However, in the NMIBC cell lines RT4 and RT112 higher expressions of ABCG2 were seen (Figure S2).

### ABCG2 is Significantly Enriched within NMIBC SP Cells and Maintains this Phenotype

We next focused on NMIBC disease and examined the relative mRNA expression of four ABC transporters (ABCA2, ABCG2, MRP1 and P-glycoprotein) in RT112 and RT4 SP and NSP fractions using real time PCR (Figure 2A and 2B).The results demonstrated that, in addition to ABCG2 being the major ABC transporter in NMIBC, its expression was higher in SP cells compared to NSP cells. The key role of ABCG2 in generating the SP phenotype was confirmed by complete loss of SP population following treatment with the ABCG2-specific inhibitor fumitremorgin C (FTC) compared to the lack of effect of treatment with the P-glycoprotein inhibitor verapamil (Figure 2C and 2D). In contrast, P-glycoprotein was the most differentially expressed and functionally relevant ABC transporter in the MIBC cell line J82 (Figure S3). Furthermore, we confirmed that in NMIBC cells the SP fraction was associated with only highest levels of ABCG2 (ABCG2^hi^) expression (Figure S4).

### SP Cells Enrich for Stem Cell Function *in vitro*

We next compared the clonogenic potential of RT112 ABCG2^hi^ SP and ABCG2^low^ NSP cells. ABCG2^hi^ SP cells displayed a three-fold increase in mean (±SE) colony forming enrichment to 56.7% (±1.6%) from 18.2% (±0.2%) in ABCG2^low^ NSP cells (Figure 3A). Similar increase in ABCG2^hi^ SP clonogenicity compared with ABCG2^low^ NSP cells was demonstrated in the additional NMIBC cell line RT4 (Figure S5). Following the gating of non-viable cells using propidium iodide, cell cycle analysis revealed that a greater proportion of ABCG2^hi^ SP cells were in S-phase in comparison to ABCG2^low^ NSP cells, with mean (±SE) values of 19% (±5%) versus 5.5% (±1) for S-phase and 68% (±8%) versus 94% (±1%) for G1-phase, respectively (Figure 3B).

To consider the ability of ABCG2^hi^ SP cells to repopulate the original phenotypic spectrum of the parent RT112 cell line, we performed serial cultures and selections of SP and NSP fractions before re-staining with Hoechst 33342 and reanalysing by flow cytometry (Figure 3C). The results showed that following the first round of selection, culture and flow cytometry, cultures derived from both SP and NSP cells each gave rise again to a similar distribution of SP and NSP cells. If this process was then continued with repeated cycles of selection, separate culture and flow cytometric sorting, the proportion of cells from SP sub-cultures that were located in the SP fraction increased with each round, whilst there was a marked decrease in the SP fraction of cells derived from NSP sub-cultures.

We assessed stem cell marker expression in RT112 ABCG2^hi^ SP and ABCG2^low^ NSP cells by real time PCR (Figure S6A). ABCG2^hi^ SP cells showed increased expression of Nanog, Notch1 and SOX2 compared to ABCG2^low^ NSP cells. Additionally, expression of bladder basal cell markers, including CK14, CD44, CD49f (α6 integrin) and CD104 (β4 integrin) also associated with enrichment of CSCs, was investigated (Figure S6B). All basal cell markers were expressed in ABCG2^hi^ SP cells but similar levels of expression were also seen in ABCG2^low^ NSP cells.

### SP Cells Enrich for Greater Tumour Initiating Ability *in vivo*

To investigate the comparative ability of ABCG2^hi^ SP and ABCG2^low^ NSP cells to form viable tumours *in vivo,* sorted cells from each sub-population were separately injected subcutaneously into CD1 nude mice and the implant sites monitored for growth. Rapid tumour growth was observed in 5 of 5, 5 of 5, and 3 of 3 animals following implantation of 10^3^, 10^4^, and 10^5^ ABCG2^hi^ SP cells respectively. However, ABCG2^low^NSP tumour growth was exhausted by dilution of seeding numbers to 10^3^ cells whilst 5 of 5 mice engrafted with 10^3^ ABCCG2^hi^ SP cells grew tumours (Table 1). Examining median tumour volume over time revealed exponential growth within the ABCG2^hi^ SP cell inoculum group in contrast to those receiving ABCG2^low^ NSP cells (Figure 4A).

Immunohistochemical analysis of ABCG2 expression in tumours, formed following the injection of 1×10^3^ ABCG2^hi^ SP cells in comparison to the residual cell pellets (which were not associated with any active growth) harvested from mice injected with ABCG2^low^ NSP sorted RT112 cells, showed increased expression in ABCG2^hi^SP compared with ABCG2^low^NSP derived tumours (Figure 4B).

We subsequently demonstrated increased expression of ABCG2 (Figure 4C) and recognised embryonic/pluripotent stem cell markers Nanog, Notch1 and SOX2 (Figure 4D–F), which have been associated with tumorigenesis [30], using real time PCR in the tumours formed following the injection of ABCG2^hi^ SP cells compared with the residual cell pellets harvested from mice injected with ABCG2^low^ NSP cells. These *in vivo* results were consistent with those obtained with the parent *in vitro* cultured cells (Figure S6A) with high expression of stem cell markers maintained in tumours originating from ABCG2^hi^ SP cells contrasting with low expression in residual ABCG2^low^ NSP. Of note, the selection of tumour initiating cells by this *in vivo* assay resulted in enrichment of these stem cell markers, over baseline levels *in vitro*, where differences were 11-fold, 80-fold, 44-fold and 140-fold for ABCG2, Nanog, Notch1 and SOX2 expression, respectively (p<0.05). Furthermore, increased Nanog, Notch1 and SOX2 expression by the ABCG2^hi^ SP mouse xenografts was also observed by immunohistochemical analysis (Figure 4G).

### Inhibition of ERK Signalling Attenuates the SP Cancer Stem Cell Function

We investigated the potential role of MAPK in the regulation of the SP phenotype in NMIBC RT112 cells. Expression of ERK, JNK and p38 as well as the PI3K/Akt and STAT signalling pathways was determined by real time PCR (Figure S7A). EGFR, ERK1 and ERK2 expression by both ABCG2^hi^ SP and ABCG2^low^ NSP cells was documented at much higher levels than the other signalling components, suggesting that these enzymes were likely to play a significant role in the MAPK signalling in RT112 cells. Enzymatic activation was subsequently explored and pERK was confirmed to be constitutively expressed at a higher level in ABCG2^hi^ SP cells (Figure 5A). Furthermore, elevated pERK expression was also seen in the ABCG2^hi^ SP xenografts (Figure S7B). To further test the relevance of the MAPK/ERK pathway as a potential target in RT112 ABCG2^hi^ SP, the functional effects of U0126, a specific inhibitor for MEK1 and MEK2, were assessed. Cells pre-treated with U0126 for 30, 60 or 120 minutes had no detectable phosphorylated ERK expression, thus confirming the inhibition of ERK activation even in the presence of EGF stimulation (Figure S7C). Additionally, there was no significant change in PI positive cells with treatment of U0126 (p = 0.38) following staining with Hoechst 33342 (Figure S7D). We subdivided the ABCG2^hi^SP fraction into cells located at the top of the tail and cells located at the end of the tail (Figure 5B), as it has been reported that cells at the tip of the SP tail, which have the highest Hoechst 33342 efflux capacity (Hoechst^low^), highly enriched the stem cell population [31]. Treatment with the MEK inhibitor had a marked inhibitory effect on the cells located in the tail end of the ABCG2^hi^SP, reducing the ABCG2^hi^SP by 2.5-fold (5.8% to 2.3%). In contrast, the inhibitor only reduced the ABCG2^hi^SP in the non-tail end by 1.5-fold (2.8% to 1.9%). These data show that U0126 is also exerting its effect on ABCG2^hi^ Hoechst^low^ cells. Treatment with U0126 also caused a dose-dependent inhibition of CFE in RT112 ABCG2^hi^ SP and ABCG2^low^ NSP cells (Figure S7E). As we have shown that ABCG2^hi^ SP cells can give rise to both SP and NSP cells (Figure S7F) and ABCG2^low^ NSP cells have very low levels of pERK, the suppression of ABCG2^low^ NSP cell CFE appears to be indirect through the effects of U0126 on ABCG2^hi^ SP cells. Of note, preferential inhibition of larger colonies, more indicative of CSC growth, was seen as the presence of colonies ≥3mm was inhibited altogether at 10^−6^ M (Figure 5C). These data show that the ABCG2^hi^ SP fraction is, at least in part, maintained by the MAPK/ERK pathway which in turn we have shown to maintain the ABCG2^low^ NSP cells.

### Expression of ABCG2 in Clinical Bladder Cancer Correlates with Grade, Stage, Recurrence and Progression Free Survival and pERK Expression

To determine the clinical significance of our *in vitro* and *in vivo* cell line findings, we investigated the expression of ABCG2 in sections of human bladder cancer. Tissue sections from a panel of 148 NMIBC (Table S2– illustrates clinical features) were processed for immunoreactivity to ABCG2 (Figure 6A). ABCG2 positivity was detected in 143 (97%) cases, and for comparison a section of normal urothelium is included which demonstrates a greater intensity of basal ABCG2 expression, which is in keeping with the emerging evidence for a basal stem cell [32]. Average immunostaining intensity scores for ABCG2 were shown to be associated with early invasive stage, pTa (n = 87) versus pT1 (n = 61), and increasing tumour grade, low grade (n = 93) versus high grade (n = 55) (p<0.05) (Figure 6B). Median follow up was for 59 months (inter-quartile range  = 48.3–96.3 months), and 31 cases (21%) showed disease recurrence and 23 cases (16%) showed disease progression. Correlations with time to recurrence and progression free survival (PFS) revealed worse outcomes associated with increasing ABCG2 expression as illustrated with Kaplan-Meier curves and Log-Rank analyses (p<0.001) (Figure 6C). These correlations remained statistically significant when stratified for grade, stage and presence of CIS (p<0.001). Using Cox multivariate analysis, which included the variables of grade, stage and intensity of ABCG2 immunostaining, revealed that ABCG2 expression remained an independent predictor of disease progression (p<0.001; RR  = 5.1; 95%CI  = 2.6–9.9). Additionally, increasing pERK expression was also associated with worse outcomes (Log-Rank p = 0.001) (Figure 6D). Furthermore, a positive correlation was demonstrated between ABCG2 and pERK expression (p = 0.005, Pearson’s r = 0.99) (Figure 6E).

## Discussion

Recent advances in the isolation and characterisation of putative CSCs have provided exciting insights into the basis of carcinogenesis and led to increasing optimism for the development of more effectively targeted cancer therapy. Studies have presented compelling data demonstrating that a number of human cancers follow the CSC model, whereby a small subpopulation of cells within a tumour is capable of tumour initiation and responsible for tumour growth and recurrence [8], [9]. The existence of cells with increased tumour initiating potential has recently been described in human bladder cancer [33]–[35] and SP has been demonstrated in bladder cancer patient samples [19]. In this study, we characterised bladder CSCs using SP and explored their modulation by the MAPK/ERK signalling pathway.

In search of a CSC hierarchy in human bladder cancer, our *in vitro* studies showed ABCG2^hi^ SP cells displayed a three-fold increase in colony forming efficiency. These data were further supported by our *in vivo* studies whereby ABCG2^hi^ SP cells were tumour initiating in comparison to ABCG2^low^ NSP cells which displayed loss of tumour growth at 1×10^3^ cell dilution. These observations highlight enrichment of CSCs within the ABCG2^hi^ SP selections. Subsequent serial transplantations would further support a hierarchical CSC relationship in these tumours. However, we have taken an analogous approach by performing serial selections and *in vitro* cultures of ABCG2^hi^ SP and ABCG2^low^ NSP cultures demonstrated the ability of ABCG2^hi^ SP cells to repopulate the original phenotypic spectrum of the parent RT112 cell line. Serial selections of ABCG2^low^ NSP cells showed weakening in the potential to regenerate both populations indicating dilution of the ability to select or generate CSCs.

ABCG2 has been shown to be the main molecular determinant of the SP phenotype [26]. Indeed, we found the SP of NMIBC RT112 and RT4 cells had higher ABCG2 mRNA expression, was completely abrogated by ABCG2-specific inhibitor FTC and contained the top 5% of ABCG2^hi^ expressing cells. Correlation to clinical NMIBC outcomes revealed ABCG2^hi^ immunostaining to be associated with worse clinical outcomes of cancer recurrence and progression, indicating a role for CSCs in bladder cancer development. Interestingly, the SP of MIBC J82 cell line had higher P-glycoprotein expression and was inhibited by verapamil, a P-glycoprotein-specific inhibitor. P-glycoprotein mRNA levels have been found to be elevated in high grade bladder tumours [36]. Additionally, the molecular pathology of NMIBC is distinct from MIBC [20]. These findings may account for the differences seen in the % of SP cells in the NMIBC and MIBC cell lines and suggest a possible switch of CSC phenotype in MIBC.

The existence of CSCs in human bladder cancer has been reported based on the use of SP, aldehyde dehydrogenase activity assay, cell surface and cytokeratin markers generally suggesting basal markers as CSC markers [19], [33]–[35], [37], [38]. Transcripts of bladder basal cell markers CK14, CD44, CD49f and CD104 were all expressed in ABCG2^hi^ SP cells but similar levels of expression were also seen in ABCG2^low^ NSP cells. Further investigation of protein expression is required. Of note, we showed protein expression of ABCG2, the main mediator of SP, is basal consistent with published data pointing towards basal markers as CSC markers. Embryonic/pluripotent stem cell-like signatures are found to be over-expressed in poorly differentiated human cancers, including bladder cancers [30]. We show that ABCG2^hi^ SP cells exhibited higher expression levels of Nanog, Notch1 and SOX2 in comparison to ABCG2^low^ NSP cells. The expression of these pluripotent stem cell factors may contribute to greater multipotency in generating SP and NSP from the ABCG2^hi^ SP cells. Interestingly, increased expression of Nanog in NMIBC CSCs has also been shown to correlate with overexpression of self-renewing marker Bmi-1 and a larger population of SP cells [39]. Furthermore, our cell cycle studies showed a greater proportion of ABCG2^hi^ SP cells were in S-phase in contrast to the G1 positioning of ABCG2^low^ NSP cells, which is consistent with Nanog, SOX2 and Oct4 in inducing longer S-phase and a shorter G1 [40], [41] . The data presented highlights a potential mechanism for the ESC-like signatures in promoting tumorigenicity and the function of these genes in bladder cancer represents an important area for further study.

Similarly to observations reported in colon cancer cells, we found inhibition of the MAPK/ERK signalling pathway by MEK1/2-specific inhibitor U0126 to reduce SP, possibly through altering ABCG2 expression or localisation [42]. MEK inhibition has also been shown to down-regulate ABCG2 expression by promoting its degradation [27] as well as downregulating a cisplatin-induced SP that is highly tumorigenic and overexpresses Nanog [43].Additionally, our data showed that inhibition of MAPK/ERK signalling pathway targeted both bladder CSCs and non-CSCs as demonstrated by reduced colony forming ability of ABCG2^hi^ SP and ABCG2^low^ NSP cells. However, the effect of U0126 on ABCG2^low^ NSP cells appears to be indirect as ABCG2^low^ NSP cells have very low levels of active ERK but are hierarchically dependent on ABCG2^hi^ SP cells with high ERK activity that would be more susceptible to U0126 inhibition. We show that ABCG2 expression correlated with NMIBC recurrence and progression, and that ABCG2 expression is, in part, regulated by MAPK activity and this correlates with ours and published findings of activated ERK predicting poor prognosis in bladder cancer [44]. Additionally, we demonstrate ABCG2 expression to positively correlate with pERK expression. Indeed, the MAPK/ERK signalling pathway has been distinctively shown to have a role in CSC self-renewal and tumourigenicity in a number of cancers [45], [46]. Our data therefore goes some way to support the MAPK pathway being central to maintenance of bladder CSC phenotype and potentially highlights strategies to usefully modulate CSCs; this approach being already a focus of preclinical and clinical studies for a number of malignancies [47]–[49]. There are encouraging results from clinical trials with MEK inhibitors [50], and our data validates the therapeutic targeting of MEK or its upstream regulators (e.g. clinical trials of erlotinib) in bladder cancer [7], [51].

In conclusion, we confirm that ABCG2^hi^ SP enriches for CSCs in human bladder cancer and provide evidence for the importance of the MAPK pathway as a suitable therapeutic target for NMIBC CSCs.

## Supporting Information

Figure S1 Gating strategy for SP data analysis. An example of the step-by-step gating strategy and the resulting percentage of cell populations is shown for RT112. (A) Gating strategy. Cells are distinguished from debris on the flow cytometric profile based on Forward Scatter (FSC) and Side Scatter (SSC) (A, 1). The Hoechst 3342 dye is excited with the UV laser at 350 nm and its fluorescence is measured with a 2-355/450/50 filter (Hoechst Blue) and a 2-355/675lp filter (Hoechst Red). Doublets and aggregates are gated out based on Hoechst Blue area versus height to ensure that a detected signal arises from single cells. PI, having been excited at 350 nm, is also measured through the 2-355/675lp filter but is much brighter than the Hoechst red signal so the dead cells line up on a vertical line to the far right (A, 2). SP cells are recognised as a distinct tail extending from the main population with the characteristic low fluorescent profile based on Hoechst Red versus Hoechst Blue (A, 3). (B) The gating tree illustrates the sequential procedure applied to select out the SP population and the percentage of cells resulting from each gating step. ‘% Parent’ indicates the percentage of gated events relative to the preceding gate. ‘% Total’ indicates the percentage of gated events relative to all events recorded.
(TIF)

Figure S2 ABC transporter expression in bladder cancer cell lines. Real time PCR was used to determine relative expression of ABC transporter mRNA in bladder cancer cell lines. Expression levels were normalised to the housekeeping gene GAPDH. Data shown are the mean of three independent experiments each done in triplicate (mean ± SE).
(TIF)

Figure S3 P-glycoprotein transporter mediates SP phenotype in J82 bladder cancer cells. (A) Real time PCR analysis of ABC transporter expression in J82 SP and NSP cell. Data shown are the mean of three independent experiments each done in triplicate (mean ± SE). *P<0.05 (Student’s two-tailed *t* -test). (B) Inhibition studies were performed on J82 cells using ABCG2-specific inhibitor fumitremorgin C (FTC) and P-glycoprotein inhibitor verapamil.
(TIF)

Figure S4 ABCG2 transporter mediates SP phenotype in RT112 and RT4 bladder cancer cells. Following staining with Hoechst 33342, cells were labelled with anti-ABCG2-FITC and normal mouse IgG-FITC was used as isotype control. (A) The 5% of cells showing the highest intensity immunofluorescence (population P2 for SP fraction and population P4 for NSP fraction) were categorised as ABCG2^hi^ population whilst the 5% of cells showing the lowest intensity of immunofluorescence (population P1 for SP fraction and population P3 for NSP fraction) were categorised as ABCG2^low^ population (representative dot plot). (B) The gating tree illustrates the sequential procedure applied to select out the ABCG2^hi^ and ABCG2^low^ populations and the percentage of cells resulting from each gating step. RT112 (C) and RT4 (D) cells labelled with anti-ABCG2-FITC and resulting ABCG2^hi^ and ABCG2^low^ populations for SP and NSP fractions. These data show that SP cells equate to ABCG2^hi^ expressing cells and the NSP equate to ABCG2^low^ expressing cells.
(TIF)

Figure S5 RT4 SP cells have a higher clonogenic ability in comparison to RT4 NSP cells. RT4 FACS sorted cells were seeded in 6-well plates at a density of 1×10^2^ cells/well and colonies were counted after 2 weeks. Data shown are the mean (± SE) of three independent experiments each done in triplicate. *P<0.05 (Student’s two-tailed *t*-test).
(TIF)

Figure S6 Stem and basal cell marker expression in ABCG2^hi^ SP and ABCG2^low^ NSP fractions of RT112 cells. Relative mRNA expression of stem (A) and basal (B) cell markers was determined in ABCG2^hi^ SP and ABCG2^low^ NSP fractions of RT112 cells using real time PCR. Data shown are the mean of three independent experiments each done in triplicate (mean ± SE). *P<0.05 (Student’s two-tailed *t* -test).
(TIF)

Figure S7 Inhibition of ERK signalling attenuates the fraction of ABCG2^hi^ SP RT112 cells. (A) Real time PCR analysis of MAPK pathway component gene expression was carried out in ABCG2^hi^ SP and ABCG2^low^ NSP sorted RT112 cells. Data shown are the mean (±SE) of three independent experiments each done in triplicate. (B) ABCG2^hi^ SP and ABCG2^low^ NSP xenografts were stained for pERK. (C) RT112 cells were starved overnight in basal media (BM) and pretreated with MEK inhibitor U0126 (10µM) for 30, 60 and 120 mins before being stimulated with recombinant EGF (r-EGF, 10 and 100 ng/ml) for 5 mins. Western blotting demonstrating pERK levels. Total ERK levels were used as loading control. (D) Cell viability using propidium iodide (PI, 2µg/ml) following staining with Hoechst 33342 in the absence or presence of U0126 (10µM) (p = 0.38, Student’s two-tailed *t*-test). (E) U0126 decreases the CFE of both ABCG2^hi^ SP and ABCG2^low^ NSP. (F) SP cells sorted from RT112 were cultured for two weeks, restained with Hoechst 33342 dye and reanalysed by flow cytometry.
(TIF)

Table S1 Primer sequences for real time PCR.
(DOCX)

Table S2 Characteristics of 148 patients with non muscle invasive bladder cancer. Table shows number of patients (%) according to age and sex, in addition to grade and stage stratified for ABCG2 immunostaining score.
(DOCX)
