# Supplementary figures and images for: Side Population in Human Non-Muscle Invasive Bladder Cancer Enriches for Cancer Stem Cells That Are Maintained by MAPK Signalling
Source: PLoS One. 2012 Nov 30;7(11):e50690. doi: 10.1371/journal.pone.0050690 (PMC3511341; doi:10.1371/journal.pone.0050690)

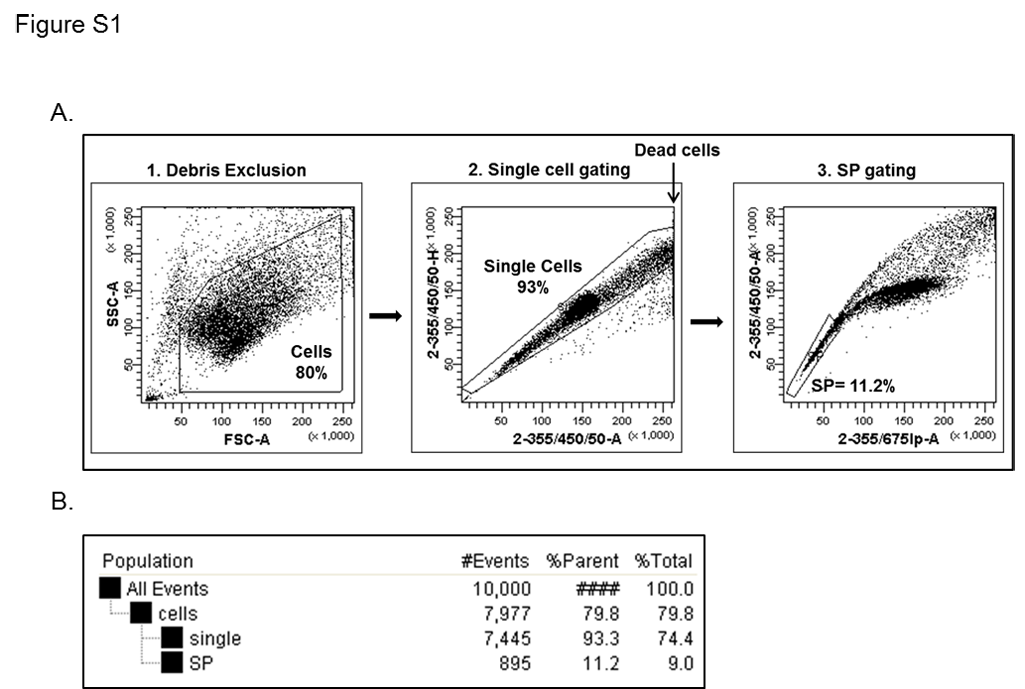

Supplement: Figure S1 — Gating strategy for SP data analysis. An example of the step-by-step gating strategy and the resulting percentage of cell populations is shown for RT112. (A) Gating strategy. Cells are distinguished from debris on the flow cytometric profile based on Forward Scatter (FSC) and Side Scatter (SSC) (A, 1). The Hoechst 3342 dye is excited with the UV laser at 350 nm and its fluorescence is measured with a 2-355/450/50 filter (Hoechst Blue) and a 2-355/675lp filter (Hoechst Red). Doublets and aggregates are gated out based on Hoechst Blue area versus height to ensure that a detected signal arises from single cells. PI, having been excited at 350 nm, is also measured through the 2-355/675lp filter but is much brighter than the Hoechst red signal so the dead cells line up on a vertical line to the far right (A, 2). SP cells are recognised as a distinct tail extending from the main population with the characteristic low fluorescent profile based on Hoechst Red versus Hoechst Blue (A, 3). (B) The gating tree illustrates the sequential procedure applied to select out the SP population and the percentage of cells resulting from each gating step. ‘% Parent’ indicates the percentage of gated events relative to the preceding gate. ‘% Total’ indicates the percentage of gated events relative to all events recorded. (TIF) [file pone.0050690.s001.tif]

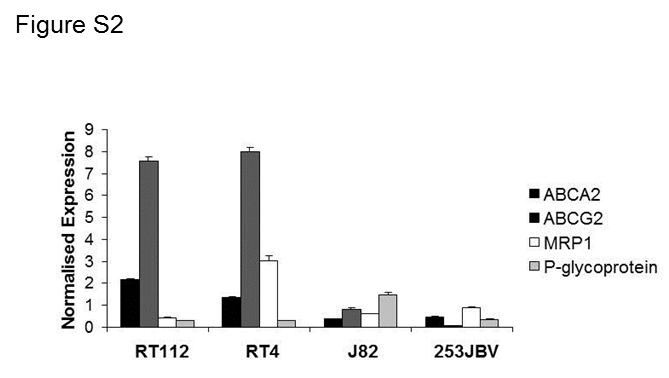

Supplement: Figure S2 — ABC transporter expression in bladder cancer cell lines. Real time PCR was used to determine relative expression of ABC transporter mRNA in bladder cancer cell lines. Expression levels were normalised to the housekeeping gene GAPDH. Data shown are the mean of three independent experiments each done in triplicate (mean ± SE). (TIF) [file pone.0050690.s002.tif]

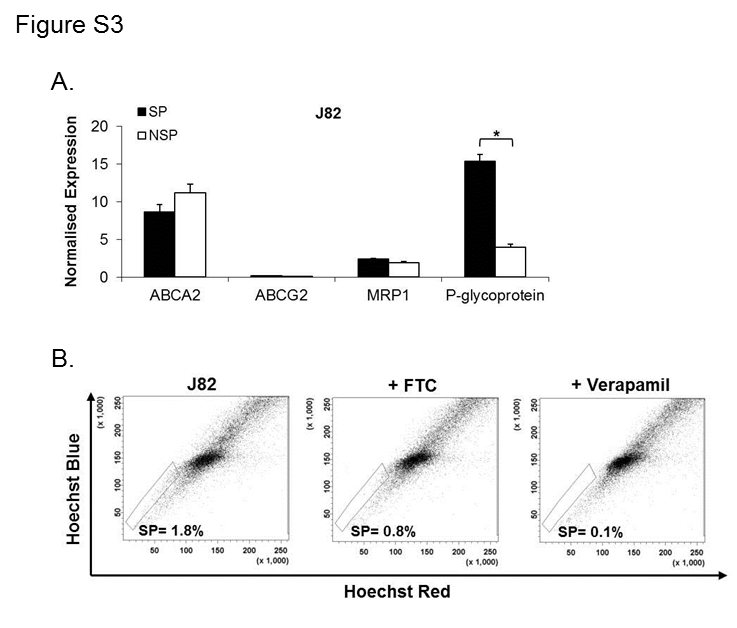

Supplement: Figure S3 — P-glycoprotein transporter mediates SP phenotype in J82 bladder cancer cells. (A) Real time PCR analysis of ABC transporter expression in J82 SP and NSP cell. Data shown are the mean of three independent experiments each done in triplicate (mean ± SE). *P<0.05 (Student’s two-tailed t -test). (B) Inhibition studies were performed on J82 cells using ABCG2-specific inhibitor fumitremorgin C (FTC) and P-glycoprotein inhibitor verapamil. (TIF) [file pone.0050690.s003.tif]

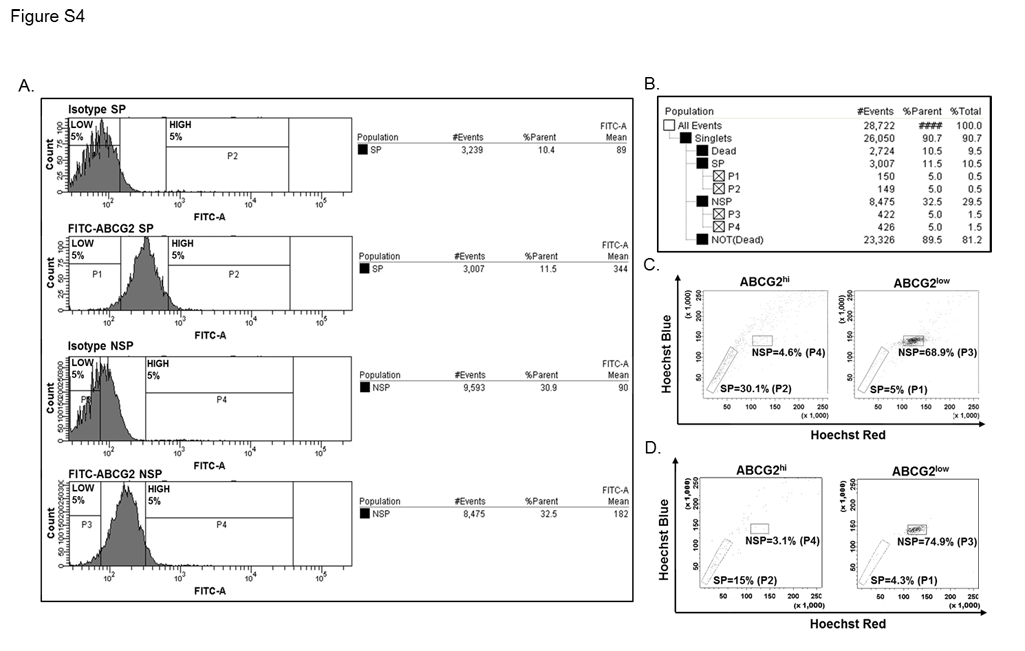

Supplement: Figure S4 — ABCG2 transporter mediates SP phenotype in RT112 and RT4 bladder cancer cells. Following staining with Hoechst 33342, cells were labelled with anti-ABCG2-FITC and normal mouse IgG-FITC was used as isotype control. (A) The 5% of cells showing the highest intensity immunofluorescence (population P2 for SP fraction and population P4 for NSP fraction) were categorised as ABCG2hi population whilst the 5% of cells showing the lowest intensity of immunofluorescence (population P1 for SP fraction and population P3 for NSP fraction) were categorised as ABCG2low population (representative dot plot). (B) The gating tree illustrates the sequential procedure applied to select out the ABCG2hi and ABCG2low populations and the percentage of cells resulting from each gating step. RT112 (C) and RT4 (D) cells labelled with anti-ABCG2-FITC and resulting ABCG2hi and ABCG2low populations for SP and NSP fractions. These data show that SP cells equate to ABCG2hi expressing cells and the NSP equate to ABCG2low expressing cells. (TIF) [file pone.0050690.s004.tif]

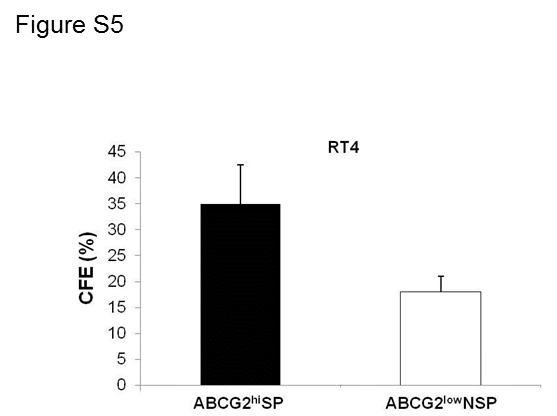

Supplement: Figure S5 — RT4 SP cells have a higher clonogenic ability in comparison to RT4 NSP cells. RT4 FACS sorted cells were seeded in 6-well plates at a density of 1×10^2^ cells/well and colonies were counted after 2 weeks. Data shown are the mean (± SE) of three independent experiments each done in triplicate. *P<0.05 (Student’s two-tailed t-test). (TIF) [file pone.0050690.s005.tif]

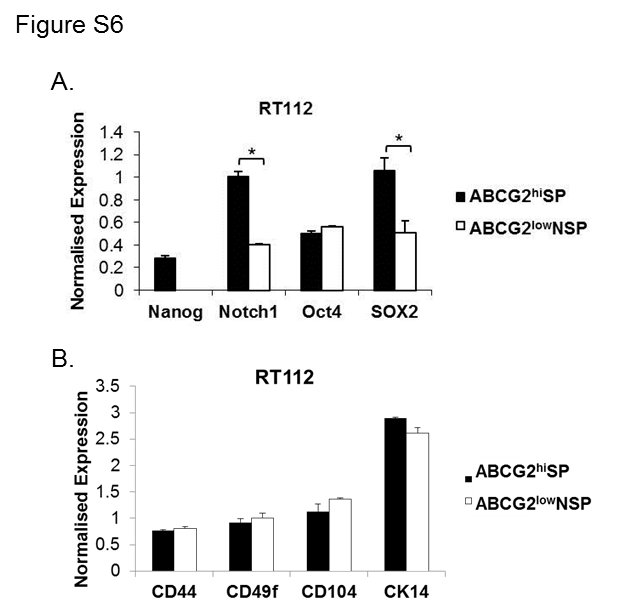

Supplement: Figure S6 — Stem and basal cell marker expression in ABCG2hi SP and ABCG2low NSP fractions of RT112 cells. Relative mRNA expression of stem (A) and basal (B) cell markers was determined in ABCG2hi SP and ABCG2low NSP fractions of RT112 cells using real time PCR. Data shown are the mean of three independent experiments each done in triplicate (mean ± SE). *P<0.05 (Student’s two-tailed t -test). (TIF) [file pone.0050690.s006.tif]

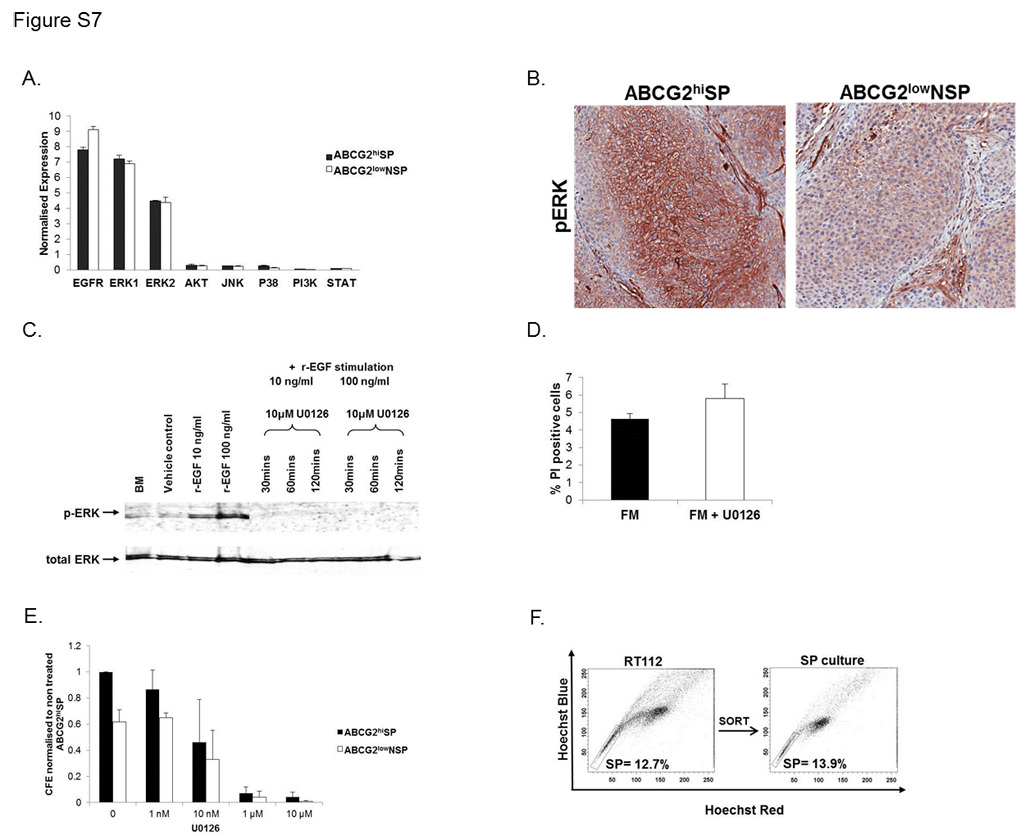

Supplement: Figure S7 — Inhibition of ERK signalling attenuates the fraction of ABCG2hi SP RT112 cells. (A) Real time PCR analysis of MAPK pathway component gene expression was carried out in ABCG2hi SP and ABCG2low NSP sorted RT112 cells. Data shown are the mean (±SE) of three independent experiments each done in triplicate. (B) ABCG2hi SP and ABCG2low NSP xenografts were stained for pERK. (C) RT112 cells were starved overnight in basal media (BM) and pretreated with MEK inhibitor U0126 (10µM) for 30, 60 and 120 mins before being stimulated with recombinant EGF (r-EGF, 10 and 100 ng/ml) for 5 mins. Western blotting demonstrating pERK levels. Total ERK levels were used as loading control. (D) Cell viability using propidium iodide (PI, 2µg/ml) following staining with Hoechst 33342 in the absence or presence of U0126 (10µM) (p = 0.38, Student’s two-tailed t-test). (E) U0126 decreases the CFE of both ABCG2hi SP and ABCG2low NSP. (F) SP cells sorted from RT112 were cultured for two weeks, restained with Hoechst 33342 dye and reanalysed by flow cytometry. (TIF) [file pone.0050690.s007.tif]
