# Supplementary material for: Side Population in Human Non-Muscle Invasive Bladder Cancer Enriches for Cancer Stem Cells That Are Maintained by MAPK Signalling
Source: PLoS One. 2012 Nov 30;7(11):e50690. doi: 10.1371/journal.pone.0050690 (PMC3511341; doi:10.1371/journal.pone.0050690)
Supplement: Table S1 — Primer sequences for real time PCR. (DOCX) [file pone.0050690.s008.docx]

**Table S1.**

| **Gene** | **Forward primer (5’-3’)** | **Reverse primer (5’-3’)** |
| --- | --- | --- |
| **ABCA2** | **CTCACTGGTGCAGACGTTGT** | **TTCAGAATGAGGTCCCAGATG** |
| **ABCG2** | **ACGGCTTTGCAGCATAATGA** | **GAGTCCTGGGCAGAAGTTTTGT** |
| **Akt** | **ACAGCCCTGAAGTACTCTTTCCA** | **CATGACAAAGCAGAGGCGGT** |
| **EGFR** | **GGTGACTCCTTCACACATACTCCTC** | **AATATCCAGTTCCTGTGGATCCA** |
| **ERK1** | **TCATGCTGAACTCCAAGGGC** | **CCACAGACCAGATGTCGATGG** |
| **ERK2** | **ACCTGCTGCTCAACACCACC** | **CCAGGCCAAAGTCACAGATCTT** |
| **GAPDH** | **CGACCACTTTGTCAAGCTCA** | **GGGTCTTACTCCTTGGAGGC** |
| **JNK** | **TTTTCCCAGCTGACTCAGAACAC** | **CCCTTGCCTGACTGGCTTTA** |
| **MRP1** | **AGCCCCCCTGCAAGTCAT** | **CCCAGATTCAGCCACAGGAG** |
| **Nanog** | **CATGAGTGTGGATCCAGCTTG** | **CCTGAATAAGCAGATCCATGG** |
| **Notch1** | **CACTGTGGGCGGGTCC** | **GTTGTATTGGTTCGGCACCAT** |
| **Oct4** | **GTCCGAGTGTGGTTCTGTA** | **CTCAGTTTGAATGCATGGGA** |
| **PI3K** | **TCAAAGGATTGGGCACTTTT** | **GCCTCGACTTGCCTATTCAG** |
| **p38** | **CTGGATGCATTACAACCAGACAG** | **ATTATGCATCCCACTGACCAAAT** |
| **P-glycoprotein** | **CCCATCATTGCAATAGCAGG** | **TGTTCAAACTTCTGCTCCTGA** |
| **SOX2** | **CCCGGCGGAAAACCAA** | **CGGGCAGCGTGTACTTATCC** |
| **STAT3** | **GCGAGGACTGAGCATCGAG** | **CCCAAGAGTTTCTCTGCCAGTG** |
