# Supplementary material for: Side Population in Human Non-Muscle Invasive Bladder Cancer Enriches for Cancer Stem Cells That Are Maintained by MAPK Signalling
Source: PLoS One. 2012 Nov 30;7(11):e50690. doi: 10.1371/journal.pone.0050690 (PMC3511341; doi:10.1371/journal.pone.0050690)
Supplement: Table S2 — Characteristics of 148 patients with non muscle invasive bladder cancer. Table shows number of patients (%) according to age and sex, in addition to grade and stage stratified for ABCG2 immunostaining score. (DOCX) [file pone.0050690.s009.docx]

**Table S2.**

| **Mean age** (range)    **Gender**  *Male*  *Female* | 70 (32-95)      116 (78%)  32 (22%) | | | |
| --- | --- | --- | --- | --- |
| **ABGC2 score** | **0** | **1** | **2** | **3** |
| **Stage** |  |  |  |  |
| *Ta* | 3 (2%) | 22 (15%) | 53 (36%) | 9 (6%) |
| *T1* | 2 (1%) | 12 (8%) | 29 (19%) | 18 (12%) |
|  |  |  |  |  |
| **Grade** |  |  |  |  |
| *Low* | 4 (3%) | 22 (15%) | 56 (38%) | 11 (7%) |
| *High (+/-CIS)* | 1 (1%) | 12 (8%) | 26 (18%) | 16 (11%) |
|  |  |  |  |  |
